# Up-to-Date Overview of the Use of Natural Ingredients in Sunscreens

**DOI:** 10.3390/ph15030372

**Published:** 2022-03-18

**Authors:** Diana I. S. P. Resende, Ana Jesus, José M. Sousa Lobo, Emília Sousa, Maria T. Cruz, Honorina Cidade, Isabel F. Almeida

**Affiliations:** 1 CIIMAR—Centro Interdisciplinar de Investigação Marinha e Ambiental, 4450-208 Matosinhos, Portugal; dresende@ff.up.pt (D.I.S.P.R.); hcidade@ff.up.pt (H.C.); 2 Laboratório de Química Orgânica e Farmacêutica, Departamento de Ciências Químicas, Faculdade de Farmácia, Universidade do Porto, 4050-313 Porto, Portugal; 3 Associate Laboratory i4HB—Institute for Health and Bioeconomy, Faculty of Pharmacy, University of Porto, 4050-313 Porto, Portugal; anaaimjesus@gmail.com (A.J.); slobo@ff.up.pt (J.M.S.L.); 4 UCIBIO—Applied Molecular Biosciences Unit, MedTech, Laboratory of Pharmaceutical Technology, Department of Drug Sciences, Faculty of Pharmacy, University of Porto, 4050-313 Porto, Portugal; 5 Faculty of Pharmacy, University of Coimbra, 3004-531 Coimbra, Portugal; trosete@ff.uc.pt; 6 Center for Neuroscience and Cell Biology, 3004-504 Coimbra, Portugal

**Keywords:** natural ingredients, sunscreens, preparations, market

## Abstract

The photoprotective skincare segment is in high demand to meet consumer concerns on UV-induced skin damage, with a recent trend towards sunscreen alternatives with a natural origin. In this study, the use of natural ingredients, either from terrestrial or marine origin, in a panel of 444 sunscreen commercial formulations (2021) was analyzed. Ingredients from terrestrial organisms represent the large majority found in the analyzed sunscreen formulations (48%), whereas marine ingredients are present only in 13% of the analyzed products. A deeper analysis regarding the most prevalent families of ingredients from terrestrial and marine organisms used as top ingredients is also presented, as well as their mechanisms of action. This study provides an up-to-date overview of the sunscreen market regarding the use of natural ingredients, which is of relevance for scientists involved in the development of new sunscreens to identify opportunities for innovation.

## 1. Introduction

Naturally occurring ultraviolet radiation (UVR) is divided into three regions, classified according to their wavelength, called UVA (315–400 nm), UVB (280–315 nm), and UVC (100–280 nm) [1]. The ozone layer acts as a filter for UVR, absorbing all UVC and 90% of UVB when sunlight passes through the atmosphere. However, some UVB and UVA radiation is not filtered by the atmosphere, reaching the Earth and the sea, and causing a harmful effect on both terrestrial and aquatic organisms [2]. Excessive solar exposure is visually characterized by the swelling and redness of the affected area, well-known as sunburn or solar erythema. Nonetheless, the deleterious effects of excessive exposure are beyond what is seen, since UVR interferes in biological and metabolic processes, triggering a cascade of reactions that cause skin photodamage, photoaging, and photocarcinogenesis [3]. These skin damage events are provoked by UVR, which alters DNA, cellular antioxidant balance, signal transduction pathways, immune system, and the extracellular matrix [4]. UVB radiation directly affects the DNA by inducing apoptosis or errors in the DNA replication, activating inflammatory processes, photo-immunosuppression, melanogenesis, and skin cancer [5,6,7]. On the other hand, DNA damage, due to overproduction of reactive oxygen species (ROS), cross-linking of collagen and elastin fibers, premature skin aging, dryness, wrinkles, hyperpigmentation, and skin sensitization are some of the consequences of excessive sunlight exposure associated with UVA radiation [5,8,9]. DNA damage is the main event that occurs at a cellular level, as a result of UVR exposure. Depending on the wavelength and energy profile of the radiation, the kind of lesions produced might be attributed to direct or oxidative DNA damage [10]. Some of these lesions can evade the endogenous DNA repair mechanisms, thus persisting and even accumulating with chronic exposure, contributing to skin photodamage [10].

Ancient evidence from paintings suggests that clothing covering the body, veils and large brim hats were used by ancient Greeks to protect themselves from solar exposure, and that umbrellas existed in ancient Egypt, Mesopotamia, China and India [11]. More recently, at the turn of the century, various plant extracts were used in folk medicine as sunscreens. One of the most effective was a chestnut extract from which aesculin was derived (1911). Later, several chemicals were introduced as UV filters, such as 2-naphthol-6,8-disulfonic acid salts (which were quite effective in both the UVB and UVA region) (1922), tannic acid (1925), benzyl salicylate (1931), para-aminobenzoic acid derivatives and 2-phenylimidazole derivatives (1942), anthranilic acid (1950), various cinnamates (1954), chloroquine (1962), benzophenones (1965) and many more since then [11]. A list containing the approved chemicals and inorganic filters that can be used in sunscreen formulations was published by the US Food and Drug Administration and the European Community [11].

Currently, general photoprotection measures include, among others, wearing protective clothing, sunglasses and a hat, seeking shade and avoiding sun exposure during peak sunlight hours, and the crucial relevance of using sunscreen [1]. Although no sunscreen is effective in reducing total UVR exposure, they are of paramount importance to minimize solar erythema, cutaneous immunosuppression, carcinogenesis, and skin aging [12]. The choice of the right sunscreen can be challenging and confusing for consumers due to the awareness of the product’s origin (either natural or synthetic) and whether they are eco-friendly and eco-sustainable. Over recent years, evidence suggesting that synthetic UVR filters may cause damage to the marine environment has emerged, eventually leading to the adoption of restrictive measures by some countries, namely to ban the sale and distribution of sunscreens containing those ingredients in certain locations (Hawaii, Key West, U.S. Virgin Islands, Palau, parts of Mexico, and the Caribbean islands) [2]. While oxybenzone has been shown to confer ecotoxicities that lead to coral reef bleaching [13,14], other UV chemical filters have been found in diverse marine organisms [14,15]. Hence, the research and development of an eco-friendly alternative is essential and might eventually lead to the reduction of the consumer’s concerns, increasing the use of sunscreens.

A recent demand for alternatives of natural origin by industries towards new consumer-oriented cosmetic formulations has led to a deeper investigation of natural sources for sunscreen application [16]. Studies reported that the addition of natural ingredients to sunscreens can increase their photoprotective properties through their antioxidant effects and the regulation of UV-induced skin inflammation, barrier impairment, and aging [16,17]. Since oxidative stress is induced by UVA through ROS, skin exposure to this radiation leads to oxidative DNA lesions [17]. The use of topical and systemic antioxidants has been explored as a means to deal with UVR-induced oxidative stress and UVA, in particular, reducing the damage caused by ROS, impeding or lessening tissue damage, and promoting repair after UVR exposure [17]. Some of these ingredients are used as extracts or come from plant extracts (tea extracts, lutein, flavonoids, fern extract, pycnogenol, and lycopene) and have been reported to protect skin against various UVR-induced damage endpoints [17]. 

Apart from the natural sources from terrestrial organisms, marine biodiversity represents an underexploited source of a wide range of naturally occurring UVR screening compounds, which can be used for cosmeceutical applications as eco-friendly and safer alternatives to synthetic UV filters [2,16,18,19]. Examples reporting algae-containing photoprotective substances (mycosporine-like amino acids (MAAs), scytonemin, sulfated polysaccharides, carotenoids, and polyphenols) [18,19,20,21,22,23] are undoubtedly the most common; however, photoprotective properties have also been described for other marine organisms like microorganisms [24], artemia [25,26,27,28,29], and plankton [30,31,32,33,34,35,36,37,38].

The aim of this work is, therefore, to analyze the use of natural ingredients in sunscreens marketed in 2021 in Europe (represented in this work by the Portuguese pharmacy market), corresponding exclusively to multinational brands.

## 2. Results and Discussion

### 2.1. Overview of the Use of Natural Ingredients in Sunscreens from Terrestrial and Marine Sources

**Figure 1:**
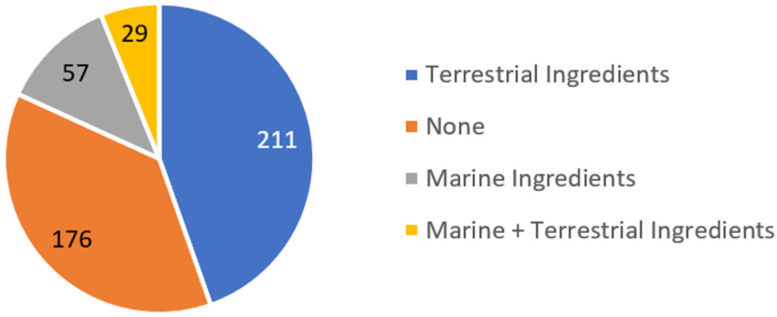
Analysis of the presence of natural ingredients from terrestrial and marine sources in the studied 444 sunscreens.

The preliminary analysis of the presence of natural ingredients in all of the studied 444 sunscreens, in a total of 43 brands, indicates that 211 (48%) contain ingredients from terrestrial organisms while marine ingredients are present in 57 (13%) of the studied formulations (Figure 1). Interestingly, only 29 (7%) of the 444 analyzed sunscreen formulations contained both terrestrial and marine ingredients and 176 (40%) did not include the referred ingredients.

According to the International Nomenclature of Cosmetic Ingredients (INCI) [39] and the glossary of common ingredient names for use on labels of cosmetic products, the classification of botanical ingredients usually requires the genus and species of the plant and comprises all products that have not undergone chemical modification including extracts, juices, waters, distillates, powders, oils, waxes, soaps, tars, gums, unsaponifiables, and resins [39]. A further distinction consists in the inclusion of the part(s) of the plant from which the material is derived [39]. 

**Figure 2:**
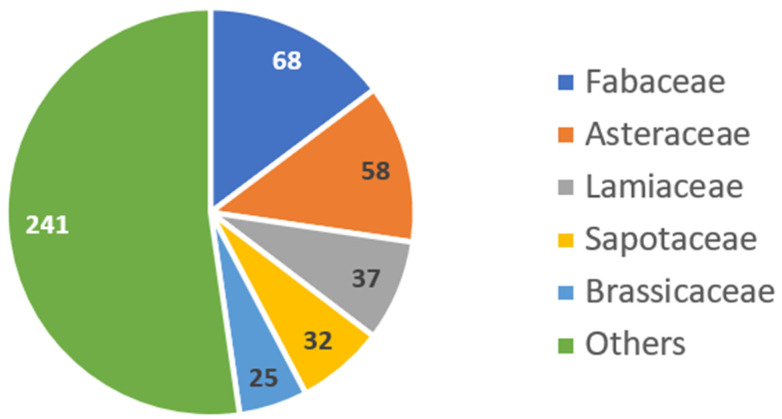
Detailed analysis on the family of natural ingredients from terrestrial sources present in the studied 444 sunscreens.

Herein, the information presented in the cosmetic product label was compiled and categorized according to the family of the specified ingredient (Figure 2). It is interesting to notice that Fabaceae, within the order *Fabales*, appears as the most relevant source of natural ingredients from terrestrial and marine sources in the analyzed sunscreens, followed by Asteraceae (Figure 2). The Fabaceae or Leguminosae, commonly known as the legume, pea, or bean family, is a large, economically and medicinally important family of flowering plants [40]. The Asteraceae family is one of the largest angiosperm families and the plants are characterized by the presence of numerous clustered inflorescences, which have the appearance of a single compound flower. It is estimated that this family represents around 10% of all flowered species, with a great biodiversity, covering all environments on the planet, except Antarctica [41]. 

**Figure 3:**
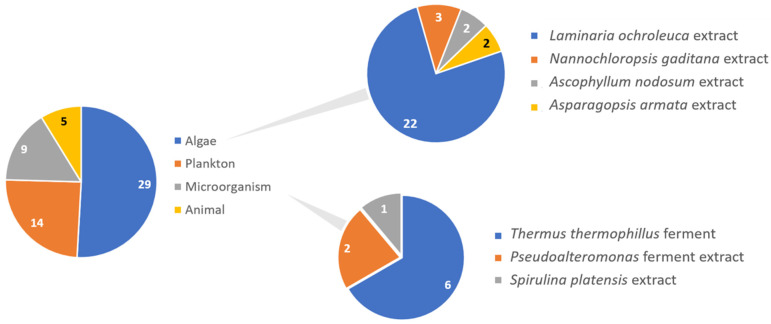
Detailed analysis on the origin of marine ingredients present in the studied 444 sunscreens.

Regarding marine ingredients, a more detailed analysis on the origin of these ingredients revealed that several types of marine organisms (algae, crustaceans, plankton, microorganisms) are used as ingredients in those formulations (Figure 3). Algae were undoubtedly the most used marine ingredient, probably due to their biodiversity, easy cultivation, and growth modulation. Brown, red, and green macroalgae account for approximately 59%, 40%, and less than 1%, respectively, of the total macroalgae cultivated in the world [42]. It is interesting to notice that the wider availability of brown and red algae is translated to their use as ingredients amongst the 444 studied sunscreen formulations, with brown algae (*Laminaria ochroleuca* and *Ascophyllum nodosum*) representing the top two used algae, followed by red algae (*Asparagopsis armata*). The latest developments concerning new methods for the production of marine organisms include more sustainable practices and allow the production/cultivation of other marine organisms besides algae (such as fish, sponges, corals, mollusks, echinoderms, *Artemia*, plankton, and microorganisms) at larger scales. However, their high potential was not translated into the sunscreen formulations containing the referred ingredients that were commercialized in 2021 in the Portuguese market and contained the referred ingredients. These ingredients were included in only 5% of the studied formulations, and comprised crustaceans, represented by *Artemia*, and *Thermus thermophilus*, *Pseudo alteromonas*, and *Spirulina platensis* representing microorganisms. 

**Figure 4:**
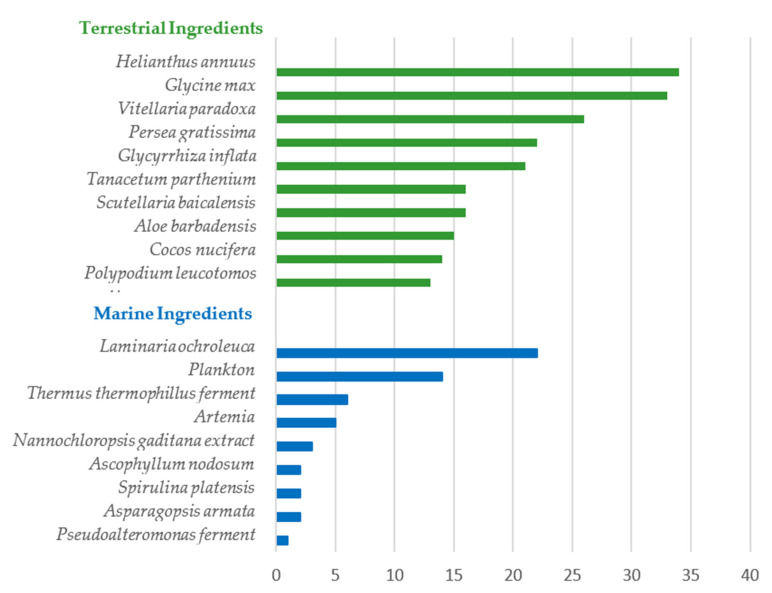
Top natural terrestrial and marine ingredients included in the composition of the analyzed sunscreen products and their relative usage in a total of 444 analyzed formulations.

To further explore the photoprotective properties of the natural ingredients included in the analyzed sunscreen formulations, the natural terrestrial and marine species with greater prevalence were selected (Figure 4). Since only nine marine ingredients were found, the top nine were considered, while for terrestrial natural ingredients, the top 10 were considered. The scientific and marketing evidence for antioxidant and photoprotective activities of these natural ingredients was compiled and will be discussed below.

There has been an enormous rise in the cosmetic market with products having a dual activity of anti-aging and sun protection. Very recently, two studies developed by us described the trends in the use of marine ingredients [43] and botanicals [44] in anti-aging cosmetics. Some of these ingredients are also present in the sunscreen products presented herein. Therefore, they were analyzed and their use in these formulations is occasionally related to their anti-aging properties instead of their photoprotective activity.

A final analysis should be made regarding the use of “non-UV filter” agents, such as extracts and other antioxidants that have been reported to add protection against exposure to UVR in sunscreens [17]. However, since all of the analyzed sunscreen formulations included one or more types of organic or inorganic filters none of the used extracts exclusively had the function of replacing the filter in the analyzed sunscreen formulations, therefore, there are no studies focused on protection against exposure to UVR. 

### 2.2. Scientific Evidence Supporting the Efficacy of the Top 10 Natural Ingredients from Terrestrial and Marine Sources Used in Sunscreens

#### 2.2.1. Natural Ingredients from Terrestrial Organisms 

Due to the variety of preparations for some botanical species, corresponding to the extraction of different parts of the plant, a categorization was performed regarding the identified natural ingredients. Differences in the plant origin, or which part of the plant or extraction method was used can lead to very diverse ingredients. Additionally, in some cases, the information found in the products’ composition list is incomplete, and for these cases, the ingredients were classified as undefined. Hence, the information presented in the cosmetic product label was compiled regarding each botanical preparation and then categorized according to the botanical species. Due to the large extension of the list of ingredients, only the top 10 botanical ingredients found in INCI lists from analyzed sunscreen products and their relative usage were analyzed regarding the scientific evidence of their photoprotective properties (Table 1).

*H. annuus*

*H. annuus* (Linné, 1753) (Asteraceae), also known as sunflower due to its rotation around the sun, is native to North America and can reach typical heights of 3 m [45]. Besides their decorative uses, sunflowers are cultivated in crops mainly for their edible fruits (also called sunflower seeds, which represent about 50% of the flower’s content) and edible oil. Since these crops are vulnerable to different atmospheric conditions, sunflowers are often cultivated in temperate climates to guarantee that their seeds are obtained in similar yields with no alterations in oil and fatty acid content [46,47]. Considering the worldwide top 3 oilseed crops (sunflower, soybean, and rapeseed), sunflower (*H. annuus*) is referred to as being one of the most profitable and providing the highest quality end products [47]. Sunflower seeds and sunflower seed oil are characterized by their high content in amino acids, tocopherols (namely α-tocopherol), flavonoids (apigenin, luteolin, quercetin, and kaempferol), phenolic acids (caffeic, caffeoylquinic, coumaric, gallic, ferulic, and sinapic), and fatty acids (eicosanoid, lauric, linoleic, linolenic, oleic, palmitic, stearic) [47,48,49]. Several medicinal applications, such as antimicrobial, antioxidant, anti-inflammatory activities, and wound-healing properties, were attributed to the use of seeds and the seed-extracted oils from sunflowers [48,50]. Additionally, these extracts also exhibited antioxidant and photoprotective activities that were associated with the presence of tocopherols, flavonoids, and phenolic acids [51,52]. Interestingly, the sunflower’s head, a by-product of sunflowers, was also reported to exhibit antioxidant activity [50].

**Figure 5:**
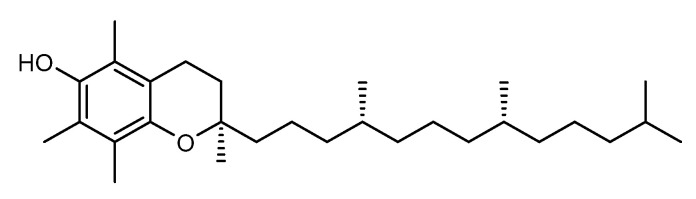
Chemical structure of vitamin E present in *Helianthus annuus* extract with skin anti-aging effects.

Naturally-occurring α-tocopherol (Figure 5), or vitamin E, is a fat-soluble antioxidant vitamin that confers protection against free radical damage, especially from ROS. Considering the excessive production of ROS derived from extrinsic factors, such as pollution, smoke, and mostly from UVR, this vitamin could play an essential role as a photo-preventive agent. Accordingly, a recent in vitro study performed by Tamara and colleagues investigated the photoprotective properties of sunflower seed oil, combined with two UV filters, oxybenzone and octyl methoxycinnamate in a cosmetic formulation [51]. The SPF value found for the formulation with 1% of sunflower seed oil, 2% of oxybenzone, and 5% of octyl methoxycinnamate was 26.03, almost 5 units higher than the cream only with the UV filters at the same percentage (SPF = 21.26) [51]. However, increasing the percentage of sunflower seed oil to 5% (SPF = 26.84) and 10% (SPF = 28.88) did not boost the SPF value as expected. For the development of a photoprotective formulation, sunflower seed oil at 5% is considered an appropriate amount to provide a good SPF value. Recently, polyphenols from *H. annuus* were introduced, leading to the cosmetic ingredient HELIOXINE^®^ being classified as a quencher of photo-induced free radical damage, by maintaining the antioxidants/ROS species equilibrium, and consequently acting as a natural preventive agent against UVR damage [53].

*G. max*

**Figure 6:**
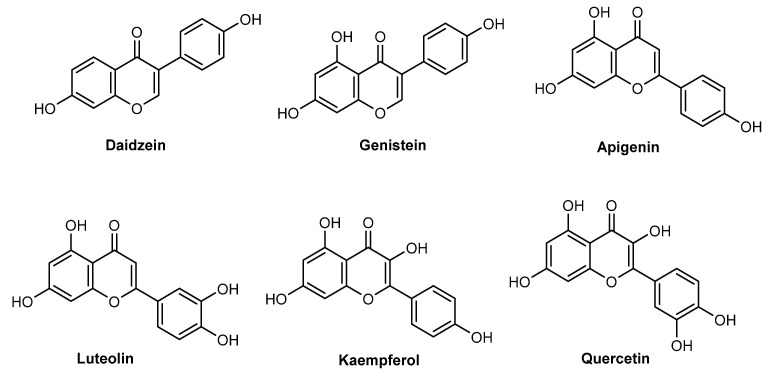
Chemical structure of compounds present in *G. max* extract with skin anti-aging effects.

*G. max* (L. Merr, 1917) oil is obtained after extraction and purification of *G. max*, a Fabaceae family’s soybean, native from China, Japan, Korea, and Russia [54]. *G. max* oil has a high content of triglycerides, such as linoleic, oleic and linolenic acids, saturated fatty acids, and flavonoids, namely isoflavones (daidzein and genistein), flavones (apigenin and luteolin), and flavonols (kaempferol and quercetin) (Figure 6) [44]. Flavonoids and phenolic acids have been widely reported due to their potent antioxidant action, as well as good absorption of UVR [55]. Quercetin is one of the natural flavonoids present in *G. max* oil and seed extracts that possess several biological activities, highlighting the strong antioxidant potential and ability to protect against UVB radiation and prevent UV-photoinduced damage in the skin [56]. Additionally, the seed extract of *G. max* is present in different cosmetics products, namely shampoo, styling products, sunscreens and anti-aging products, also due to its anti-inflammatory and antioxidant activities, and sun protection characteristics [57]. *G. max* oil also contributes to skin regeneration, stabilization, smoothing, and wetting properties required in cosmetic products [58,59,60,61].

*V. Paradoxa*

Formerly known as *B. parkii*, *V. paradoxa* (C.F. Gaertn, 1807), also commonly known as Sheatree butter, is a native from central Africa, and it is rich in triglycerides (oleic, stearic, palmitic, linoleic, and arachidic) [62]. This tree from the Sapotaceae family reaches up to 22 m in length, and a good quality shea fruit only appears after 15 years of cultivation, being the fruit’s maximum fat content present only after 30 years of growth [62]. Saponifiable matter, with oleic and stearic acids as major constituents, represents about 85–90% of the total composition, and 10% corresponds to unsaponifiable matter, such as catechins, triterpenes, tocopherol, phenols, and sterols [62,63]. Shea butter and oil were reported for their antioxidant, anti-inflammatory, UV-filtering and anti-photoaging activities [62,63,64]. The shea butter is generally used for nutritional purposes, such as in chocolate confection [62], and for cosmetic uses as a moisturizer and skin barrier-protective agent [65]. It was recently proved that shea butter improves collagen production [44] and the combination of the unsaponifiable compounds contributes to the absorption of UVR [66,67]. 

Two studies published in 2020 confirmed that *V. paradoxa* exhibits photoprotective activity and a photostabilization effect on the physical properties of a cosmetic formulation, such as colour, texture, melting point, and in vitro sunscreen effectiveness [68,69]. The studies showed an increase of in vitro SPF value when shea butter was added to a formulation containing ethylhexyl methoxycinnamate (EHMC) and titanium oxide (TiO_2_), an organic and an inorganic UV filter, respectively. Additionally, results showed the chemical stabilization of EHMC [68], a UVB filter commonly used in sunscreen formulations, known to be photounstable [70]. Two concentrations of this botanical butter (10% and 15%) were tested in a base lipstick sunscreen containing 10% of TiO_2_ and 7.5% of EHMC. The formulations were tested in healthy volunteers, with different phototypes, and using a solar simulator, mimicking real conditions of UV-exposure, with obtained SPF values of 28.7 (10% of *V. paradoxa*) and 39.1 (15% of *V. paradoxa*) [68]. The photostabilization of EHMC conferred by sheatree butter was also confirmed by high-performance liquid chromatography (HPLC). The initial concentration of EHMC found in the formulations with 10% and 15% of *V. paradoxa* were 73 µg/mL and 77 µg/mL, respectively. After UV irradiation, a decrease of 10% (65 µg/mL) and 2% (75 µg/mL) in EHMC concentration of the formulations with 10% and 15% of *V. paradoxa*, respectively, was observed [68], allowing one to ascertain that the shea butter natural ingredient, when combined with this organic UVB filter, diminished the EHMC degradation, augmenting the formulation stability to UVR effects. Additionally, shea butter ameliorates the texture of skin and makes it softer, possesses wound-healing characteristics, helps in skin tissue regeneration, and acts as a UV-absorber agent due to the presence of cinnamate esters of triterpene alcohol, known as UVB radiation absorbers [64].

*P. gratissima*

*P. gratissima* (Miller, 1768) is mainly known for its extract, oil, flowers and fruit (avocado), and it is classified as a member of the Lauraceae family [71]. This tree can reach 12–15 m in length. It originates from Mexico, but is now planted in countries with tropical and sub-tropical climates [72]. *P. gratissima* oil and fruit are mainly used for culinary uses, namely snacks, desserts, and salads, where avocado oil is considered one of the most expensive among all the edible oils [72]. The percentage of fatty acids in avocado varies significantly depending on the cultivation area, the climate, and the degree of the fruit’s maturation [71].

**Figure 7:**
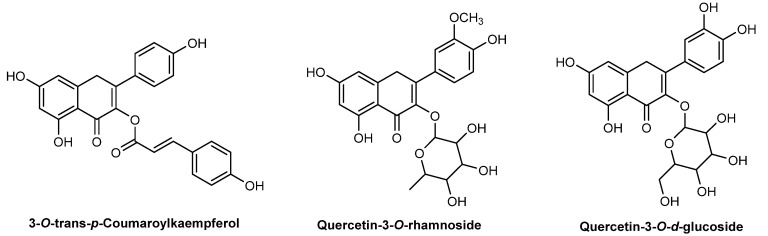
Chemical structure of compounds present in *P. gratissima* extract with skin anti-aging effects.

*P. gratissima* extracts include fatty acids (approximately 75%), namely oleic, palmitic, caproic, linoleic, margaric, 9-hydroxyoctadecanoic, and stearic (oleic and palmitic acids represent more than 50% of the total fatty acid content). Other constituents include vitamins, mineral potassium, phytosterols, and carotenoids [72,73]. Three flavonoids with potential therapeutic applications, namely antioxidant, antitumor, antimicrobial, and anti-inflammatory activities, were identified in the flowers and seeds of *P. gratissima*: 3-*O*-*trans*-*p*-coumaroylkaempferol, quercetin 3-*O*-rhamnoside, and isorhamnetin 3-*O*-*d*-glucoside (Figure 7) [74]. *P. gratissima* oil extract is widely used in different cosmetic products, cleansing products, hair conditioners, lipsticks, sunscreen agents, and moisturizers [75]. Until now, only one study was published demonstrating the photoprotective action of *P. gratissima* extract oil sterols against the UVB-induced damage through the inhibition of pro-inflammatory mediators [76].

*G. inflata*

**Figure 8:**
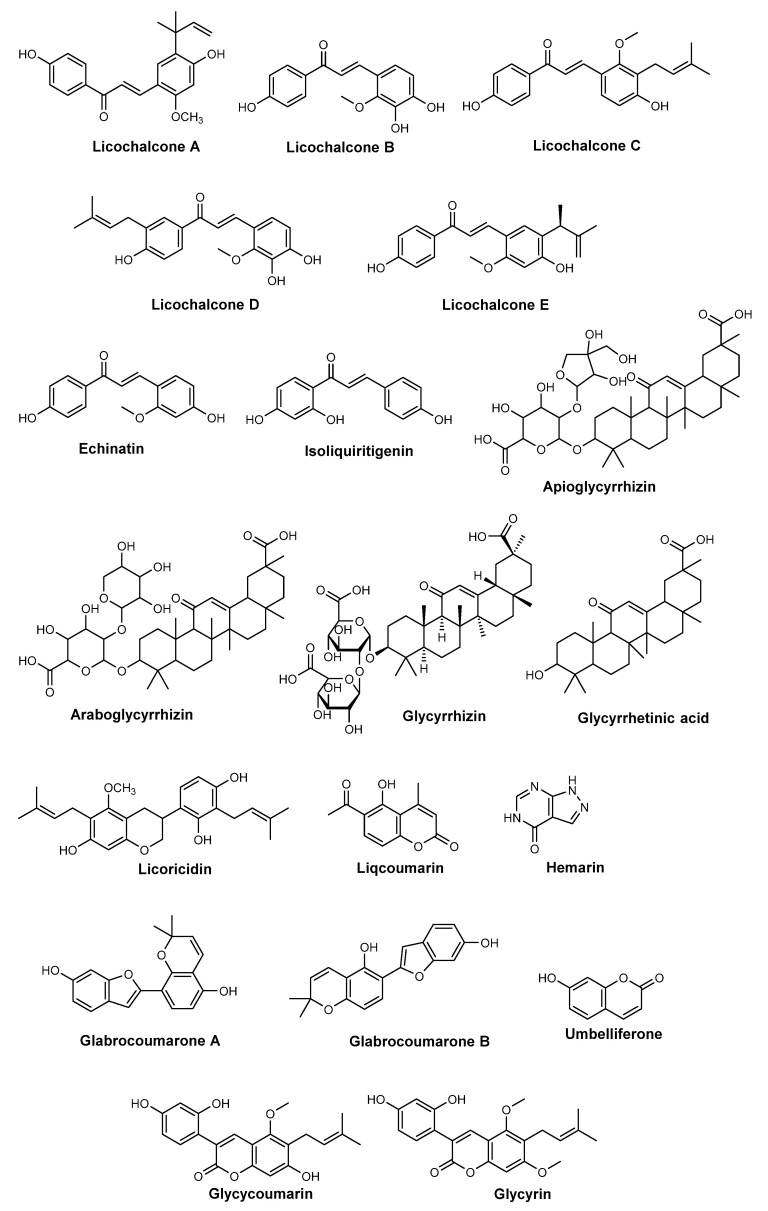
Chemical structure of compounds present in *G. inflata* extracts with skin anti-aging effect.

Native from central Asia, *G. inflata* (Batalin, 1891), also known as Chinese licorice, belongs to the Fabaceae family, and its use dates back to traditional Chinese medicine [77]. Flavonoid compounds were identified by HPLC in *G. inflata* root extract, namely licochalcone A-E, echinatin, and isoliquiritigenin (Figure 8) [78,79]. These nature-derived chalcones were reported to exhibit antioxidant and anti-inflammatory activities, inhibiting the phosphorylation of transcription factor nuclear kappa B (NF-κB) [78,80]. Besides the strong content in flavonoids, *G. inflata* is also constituted by triterpenoids, such as apioglycyrrhizin, araboglycyrrhizin, glycyrrhizin, glycyrrhetinic acid, licoricidin, and inflasaponins, and coumarin derivatives, including liqcoumarin, glabrocoumarone A and B, hemarin, umbelliferone, glycyrin, and glycocoumarin (Figure 8) [77,79]. Chinese licorice has also been reported for its diverse biological activities, namely antioxidant, antiviral, antimicrobial, and anti-inflammatory activities [79,80,81]. In addition, this is one of the most common extracts used in sunscreens [82]. Evidence of the anti-aging properties of these extracts, through the inhibition of enzymes involved in collagen degradation, especially tyrosinase and elastase enzymes, was also reported [83,84]. Licoricidin, one of the triterpenoids found in *G. inflata* root extract, was tested in human fibroblasts and revealed interesting activity as a preventive agent of UVA-induced photoaging (Figure 8) [85,86].

*T. parthenium*

**Figure 9:**
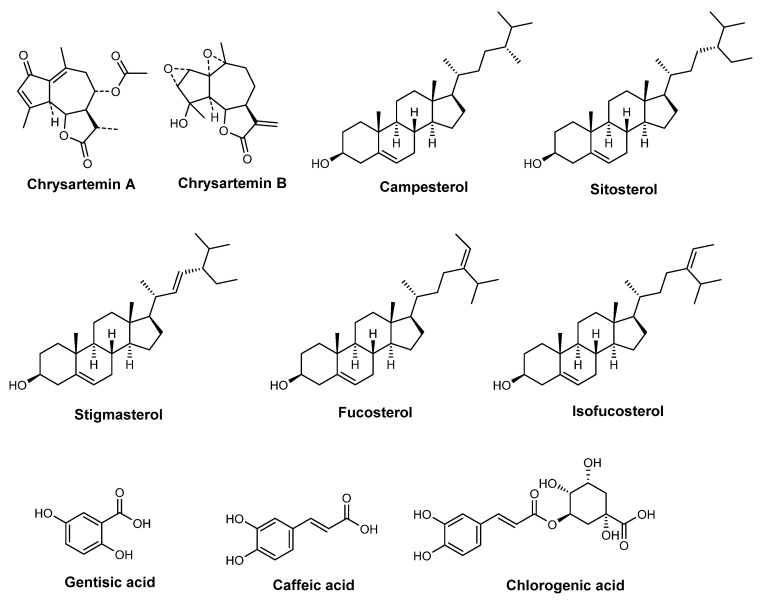
Chemical structure of compounds present in *T. parthenium* extract with skin anti-aging effects.

*T. parthenium*, with synonym *C. parthenium* (feverfew) (Schultz_Bip., 1844), is a common oriental flower from the Asteraceae family, classified as a small plant that does not grow more than 50 cm [87]. Native to limiting countries within Europe and Asia, nowadays it is widely dispersed around the world [87]. *T. parthenium* is mainly used as an ornament plant, in traditional medicine, and nutrition, especially its flower-head, leaves, and stem [88]. Feverfew was already reported as possessing several biological activities, namely, antibacterial, anti-inflammatory, antioxidant, antiviral activities, and photoprotective, neuroprotective, analgesic, antipyretic, and immunomodulatory properties [86,88]. Essential oils (oxygenated monoterpenes, monoterpene hydrocarbons, and oxygenated sesquiterpenes) [88], terpenes-lactone compounds (chrysartemins A and B) [89], sterols (campesterol and sitosterol (80%), stigmasterol (15%), and stanols, fucosterol, and isofucosterol (5%)) [90], phenolic acids (gentisic, caffeic, and chlorogenic acids) [91], and flavonoids (luteonin, quercetin, apigenin, and flavonol 6-hydroxykaempferol-3,7,4’-trimethyl ether) (Figure 6) [91,92] are the major constituents of *T. parthenium* extracts (Figure 9).

Polyphenolic compounds, such as phenolic acids and flavonoids, are well-known for their antioxidant [91] and anti-inflammatory [92] activities. Phenolic acids (gentisic, caffeic, and chlorogenic acids) and flavonoids (luteolin), were found in a concentration inferior to 0.2 µg/g plant extract, except quercetin (Figure 6) (27.61 ± 0.39 µg/g plant extract) and apigenin (9.71± 0.18 µg/g plant extract) [91]. The evaluation of the 2,2-diphenyl-1-picrylhydrazyl (DPPH) scavenging activity of *T. parthenium* extract allowed researchers to verify that this product showed a weak antioxidant activity (IC_50_ value of 149.76 ± 6.23 µg/mL), when compared to quercetin used as a positive control (IC_50_ = 5.47 µg/mL) [91]. 

Some *Tanacetum* species extracts revealed potential to act as a preventive agent of UVB-induced skin damage [93]. *T. parthenium* extract showed maximum absorption in the UVB region of the UV spectrum along with a reduction of UVB-induced expression of metalloproteinases (MMPs) involved in the photo-induced inflammatory processes, and the maintenance of the antioxidant/ROS species levels in the skin, revealing an ability to prevent and treat photo-induced skin damage [93], sound evidence of the photoprotective potential of this botanical species.

*S. baicalensis*

**Figure 10:**
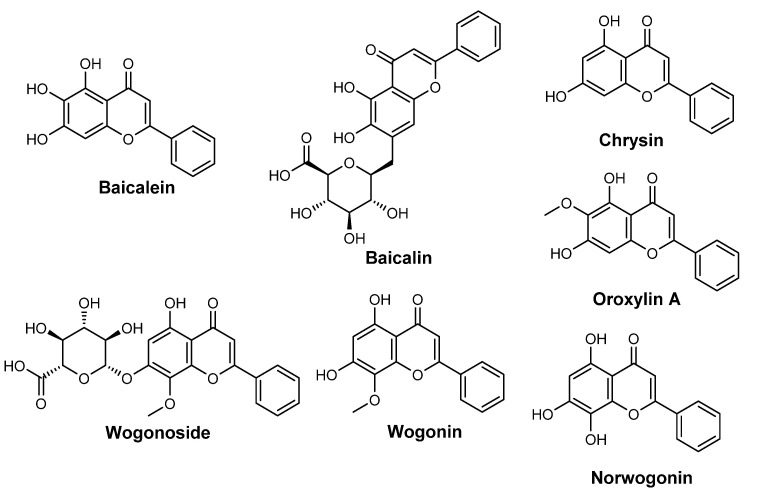
Chemical structure of compounds present in *S. baicalensis* extract.

*S. baicalensis* (Georgi, 1775) (Laminaceae), also known as Chinese skullcap, is a flowering plant native to Asian countries. It has been used for two thousand years for medicinal purposes due to its diverse array of biological activities. *S. baicalensis* possess clinical applications such as antioxidant, antitumor, antiviral and antibacterial activities, and hepato- and neuroprotective effects [94]. Its high content in flavonoids, more than 30 metabolites, made this extract a source of several active molecules. Chinese skullcap extract also includes amino acids, essential oils, sterols, and phenolic compounds. All of the constituents confer the colour to the flowers (pigmentation) and photoprotection against UV-induced damage [94]. The major flavones present in the *S. baicalensis* root extract are baicalin, baicalein, wogonoside, wogonin, norwogonin, oroxylin A, and chrysin (Figure 10) [94].

Considering the high potential of flavonoids as strong antioxidants and absorbers of UVA and UVB radiation, the root extract of *S. baicalensis* was introduced in cosmetic formulations and evaluated regarding its photoprotective potential [95]. The sunscreen formulation with zinc oxide (ZnO) was tested with and without *S. baicalensis* root extract, and the SPF value was assessed. The formulation containing ZnO and 5% of the natural extract revealed a higher SPF value (22.7), when compared with the formulation only with ZnO (SPF = 17.8), showing its photoprotective potential to act as a sunscreen agent [95]. Additionally, another study reported in 2013, confirmed the photoprotective characteristics of cosmetic formulations that contain baicalein or *S. baicalensis* extract with the same percentage of this flavonoid, attributed to their UVA filtering capability [96].

*A. barbadensis*

**Figure 11:**
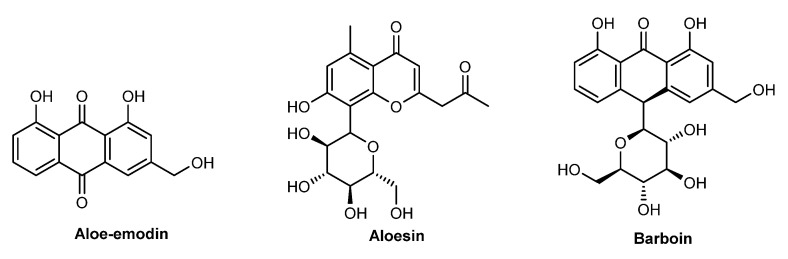
Chemical structure of compounds present in *A. barbadensis* extract with skin anti-aging effects.

More than 500 species constitute the Aloe family, and *A. barbadensis* (Miller, 1768), usually known as *Aloe vera* (Asphodelaceae), is one of the most popular botanical species belonging to this genus [97]. Native from the Arabian Peninsula, it is cultivated in tropical and semi-tropical climates, and arid soils worldwide, reaching up to only 1 m in length. *A. barbadensis* is widely used for medicinal, decorative, and cosmetic purposes, including a wide variety of products, namely lotions, creams, gels, and ointments. The *A. barbadensis* extract or juice extract is contained in the leaves of the *Aloe vera* plant. Its composition is rich in mono- and polysaccharides [98], namely mannans, polymannans, terpenoids, barbaloin, anthraquinones such as aloe-emodin, chromones such as aloesin, amino acids, minerals and vitamins B, C, and E (Figure 11) [83,86]. *A. vera* demonstrated a wide array of biological activities including anti-aging, skin-regeneration, collagen-stimulation, anti-inflammatory, antibacterial, and antioxidant activities and moisturizing and emollient properties [83,86]. The use of *A. barbadensis* in creams offers moisturizing effects, and prevention against photo-induced aging [83], and of UV-induced skin damage, such as photo-immunosuppression [99]. Several studies confirm the photoprotective potential of *A. barbadensis* extract [98,100,101,102,103]. In 2016, a study by Rodrigues et al. showed that *A. vera* extract protects keratinocytes from UVA-induced oxidative effects, suppresses UVA-induced autophagy, and reduces the loss of membrane integrity after photo-induced damage [98]. Later, in 2020, Rodrigues et al. developed a solid lipid nanoparticle-containing *A. barbadensis* extract, evaluating its photoprotective potential. Interestingly, the SPF values obtained, either in in vitro or in vivo assays, were similar (16.9 ± 2.44 and 14.81 ± 3.81, respectively) [104].

*C. nucifera*

*C. nucifera* (Linnaeus, 1753) or coconut tree belongs to the Arecaceae family, well-characterized for its palm leaves, and is usually found in coastal areas associated with tropical regions, reaching up to 30 m in length [105]. Its juice or water is appreciated by tourists when they visit tropical and subtropical areas, and its origin is related to Pacific Islands in southeast Asia, such as the Philippines, Malaysia, and Indonesia [105]. The fruit is rich in fatty acids (caprylic, capric, lauric, myristic, palmitic, and oleic acids that represent almost 90% of its content), carbohydrates, proteins, and a small percentage of vitamins and minerals [105,106,107]. *C. nucifera* oil possesses some interesting activities such as antioxidant and antimicrobial activities, prevention of cardiac and Alzheimer’s diseases, due to high density of lipids that contribute to good cholesterol, and could be used to maintain the health of teeth, hair, and skin; for these reasons, it is widely used in cosmetic products [105,107,108].

At least three studies have reported the photoprotective potential of coconut oil [109,110,111]. In 2010, Kaur and Safar evaluated the in vitro SPF value of plant-derived oils typically applied in cosmetic products, where coconut oil showed an in vitro SPF value of 7.119. When compared to the other 13 vegetable oils, coconut oil presented one of the highest SPF values [109]. In 2017, Widiyati investigated the photoprotective action of this oil in a cosmetic cream, which only revealed absorption in the UVC region of the UV spectrum and insignificant absorption of UVA and UVB radiation. However, the combination of coconut oil with UV filters that absorb in the UVA and UVB regions, such as titanium oxide (TiO_2_) and benzophenone-3, was demonstrated to be favorable, having the cream that contained benzophenone-3 (UVA filter) and coconut oil with protective action against UVB and UVA radiation [110]. Oliveira et al. quantified the phenolic composition of *C. nucifera* extract and evaluated its antioxidant and photoprotective activities, either isolated or incorporated in a formulation [111]. Quercetin (Figure 6), (+)-catechin, vanillic and caffeic acids, and (−)-epicatechin were detected and quantified by HPLC-MS analysis of the extract. All of the phenolic compounds were previously reported to exhibit antioxidant activity [111,112]. Regarding the photoprotective activity of the cream containing the *C. nucifera* extract and the cream containing quercetin, the SPF values varied between 5.0–14.09 and 1.44–15.04, respectively. This study highlighted the potential of this natural product in cosmetic formulations, as an extract or with only one ingredient, to be used as a sustainable alternative to be incorporated in cosmetic formulations [111].

*P. leucotomos*

*P. leucotomos* (Linnaeus, 1753), a native from South America, belongs to the Polypodiaceae family, and is classified as a tropical fern [113,114]. Several studies reported its medicinal applications in diminishing UV-induced damage and inflammatory and oxidative processes, as well as its use in some skin disorders, namely solar erythema, melasma, and atopic dermatitis [113,114,115,116], and contributing to fight skin photo-aging [83]. *P. leucotomos* extract is mainly constituted by phenolic compounds, namely phenolic acids (benzoic, cinnamic, caffeic, ferulic, coumaric, among others acids) and monosaccharides [117]. Phenolic acids offer antioxidant and photoprotective activities, and also contribute to the decrease of UV-induced inflammation and oxidative damage [83]. González and Pathak (1996) demonstrated the protective effect of this botanical extract against UVA- and UVB-induced damage, by decreasing the formation of ROS, lipid peroxidation and photosensitization, following either topical application or oral administration [118]. *P. leucotomos* extract was also reported to inhibit tumour necrosis factor alpha (TNF-α), and the production of cellular nitric oxide (NO) through the inhibition of nitric oxide synthase (iNOS), in conditions that mimic the solar exposure-induced damage [119], and for protecting epidermal keratinocytes and dermal fibroblasts exposed to UV radiation [120].

#### 2.2.2. Marine Ingredients

*L. ochroleuca*

**Figure 12:**
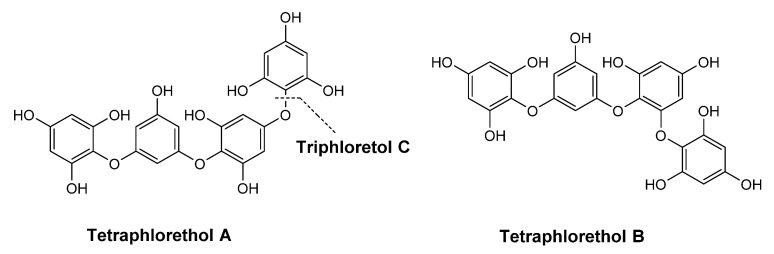
Structures of triphlorethol C and tetraphlorethols A and B extracted from *L. ochroleuca*.

*L. ochroleuca* (Bachelot de la Pylaie, 1824) is a yellow-brown digitate kelp that reaches up to about 1.5 m in length. Widely consumed in Asian countries, this edible alga is mainly valued in Europe through its extracts, which include alginates, bioactives, and pigments [121]. While alginates are usually commercialized to be used as thickening, gelling, and stabilizing agents in food, cosmetic and pharmaceutical industries, this alga also contains other bioactives, such as fucoidans that reduce the expression of the pro-inflammatory cytokines and have antioxidant, antimicrobial, and antitumor properties [122], and phenolic compounds to which are usually associated antioxidant properties [20,123,124]. An example of phenolic compounds identified in extracts of *L. ochroleuca* is the linear phlorethols, which may have *ortho*-, *meta*- or *para*-oriented biphenyl ether bridges or combinations such as triphlorethol C and acetylated tetraphlorethols A and B (Figure 12) [125,126].

The study of the seasonal and yearly variations in phenolic contents and associated cosmetic activities of seven brown marine macroalgae, including *L. ochroleuca*, revealed that the phlorotannin content of the studied algae was high regardless of the season, and antioxidant and photoprotective activities were similar to those of commercial molecules (butylated hydroxyanisole (BHA) and vitamin C) [20]. Concerning *L. ochroleuca* in particular, no statistically significant variation was observed (*p* > 0.05) between the seasons (autumn, winter, and spring), although seaweeds harvested in autumn appeared to have the highest radical scavenging activity in the DPPH test and antioxidant activity on the ferric reducing antioxidant power (FRAP) assay [20]. The phlorotannin enriched fraction of *L. ochroleuca* also revealed potential photoprotective activities since the photoprotective factors were greater than 1 (between 1.24 and 2.15 for sun protection factor (SPF) and between 1.19 and 1.96 for UVA protection factor (PF-UVA)), with no seasonal differences [20].

Plankton

The planktonic compartment of marine resources, composed of zooplankton, phytoplankton, bacteria, and viruses, represents 95% of marine biomass [127]. Although the potential is largely unexplored, planktonic organisms offer already immense opportunities: new resources for medicine, cosmetics, and food, renewable energy, and long-term solutions to mitigate climate change [127]. Regarding its usage in cosmetics, plankton is included in skincare compositions to improve the health and physical appearance of skin; however, there is a paucity of evidence regarding the antioxidant and photoprotective properties of plankton.

**Figure 13:**
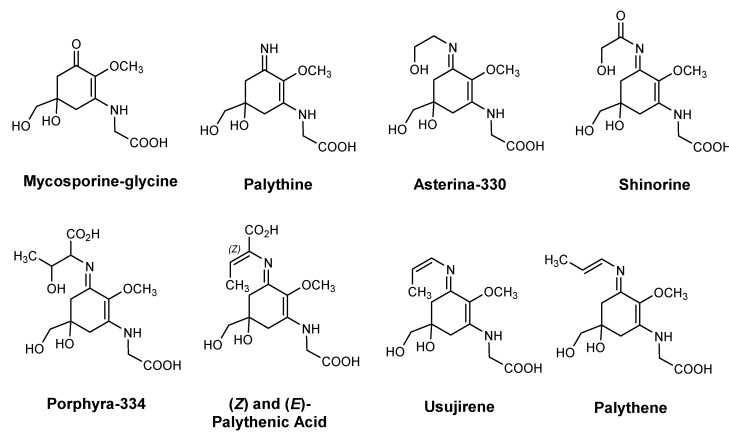
Mycosporine-like amino acids isolated from *A. excavatum* [34].

Planktonic species are exposed to solar UVR, which penetrates the water column [33]. These organisms, upon different environmental stress factors (e.g., seasonal changes in UV levels and temperature), have evolved a variety of response mechanisms to prevent or repair damage from UVR [33]. Besides regulating their position in the water column, they also have the tendency to accumulate or synthesize sunscreens (e.g., carotenoids and MAAs), or repair DNA damage (e.g., photoenzymatic repair and nucleotide excision repair) [33,38]. Specific examples of MAAs isolated from *Alexandrium excavatum*, a red-tide dinoflagellate, are represented in Figure 13 [34].

The research on the photoprotective potential of MAAs has mostly been focused on the species in which MAA compounds are produced or found [31,37,38,128]. There has been surprisingly little work carried out in skin models to demonstrate their potential for human use [129,130]. However, preliminary data strongly suggest that MAAs have the potential for the protection of human skin from a diverse range of adverse effects of solar UVR, including UVR-induced toxicity, DNA damage/erythema, inflammation, photoaging [129], and the damage induced by free radicals produced by UVR exposure through their antioxidant activity [131,132].

In another example, the antioxidant properties of aqueous extracts from the zooplankton dinophycean flagellates *Gymnodinium impudicum* and *Alexandrium affine* and the raphidophycean flagellate *Chattonella ovata* were examined [132]. All of the extracts directly scavenged superoxide and the superoxide scavenging potential of any of the extracts was comparable to that of L-ascorbic acid. As for hydroxyl radical scavenging, the Fenton reaction and the method of ultraviolet radiation to hydrogen peroxide were used as hydroxyl radical generation systems [132]. Although a reduction of the level of hydroxyl radicals in both of the systems for all the extracts was noticed, the fact that the levels of phenolic compounds exhibited a correlation with the antioxidant activities of the extracts indicates the presence of substances other than phenolic compounds that might be responsible for the activities.

Although the regular use of sunscreen has demonstrated a protective effect towards the development of skin cancer, newer options to help repair damaged DNA may have an important role in reducing the incidence of chronic sun exposure-related photoaging and non-melanoma skin cancer [10]. Plankton extract, which contains the DNA repair enzyme photolyase, is one novel ingredient that is currently being incorporated into sunscreens to complement intrinsic DNA repair, thus expanding the photoprotective abilities of sunscreens to photo repair [10]. This enzyme is capable of repairing cyclobutane dimers formed as a result of DNA UV irradiation [10].

*T. thermophillus* Ferment

Probiotics have also attracted great attention towards the development of natural non-toxic antioxidants due to their capacity to decrease the risk of accumulation of ROS and to potentially be used to reduce oxidative stress [133]. Their in vitro and in vivo antioxidant capacity can be attributed mainly to the presence of specific antioxidant enzymes, the release of different antioxidant compounds acting mainly as free-radical scavengers such as glutathione, the production of some extracellular polysaccharide biomolecules [134,135], or even the exhibition of metal chelating activity [136]. Additionally, probiotic microorganisms are known to have beneficial properties such as soothing inflammation, balancing skin microflora, and inhibiting the growth of harmful bacteria [137].

Although currently there is a paucity of data on the antioxidant activity of *T. thermophillus* ferment, a study in the field of compositions (either topical or food) revealed that these can benefit from the use of a probiotic/antioxidant composition with improved antioxidant capabilities [137]. In particular, an example of a topical composition containing 10% of a probiotic blend (≈70% α-glucan oligosaccharide, ≈19% *polyminia sonchifolia* root juice, ≈10% maltodextrin, ≈1% lactobacillus), 60% of an antioxidant blend (≈95% *T. thermophillus* ferment, ≈5% glycerin), 20% of methylsilanol hydroxyproline aspartate, and 10% of *Triticum monococcum* [wheat] seed extract, acted synergistically as an antioxidant in sufficient amount to inhibit signalling pathways that contribute to skin inflammation [137]. Additionally, specific enzymes from *T. thermophillus* can be used on damaged skin exposed to UV radiation and heat to catalyze the scavenging of free radicals caused by UV damage [138].

To provide a solution to prevent and/or treat damage associated with sun-derived infrared A (IRA) radiation of the skin and its appendages, a fermentation medium of *T. thermophilus* was developed by the cosmetic industry [139,140] and marketed as an anti-aging ingredient used in cosmetic compositions for protecting the cellular structures from damage caused not only by UV but also IR radiation. In the study that accompanied the development of this product, it was shown that it can limit the increase of the formation of pro-inflammatory mediators (PGE2, IL-6 and IL-8) and reactive oxygen species (ROS) by IRA [139]. Additionally, this product improves epidermis quality (stimulation of the synthesis of involucrin, ceramides, and/or intercellular lipids) and can limit the decrease in the synthesis of macromolecules structuring the extracellular matrix [collagen-1, fibrilin-1, and/or HSP-47 (heat shock protein)], caused by the alteration of the dermal integrity by IRA [139].

*Artemia* Extract

**Figure 14:**
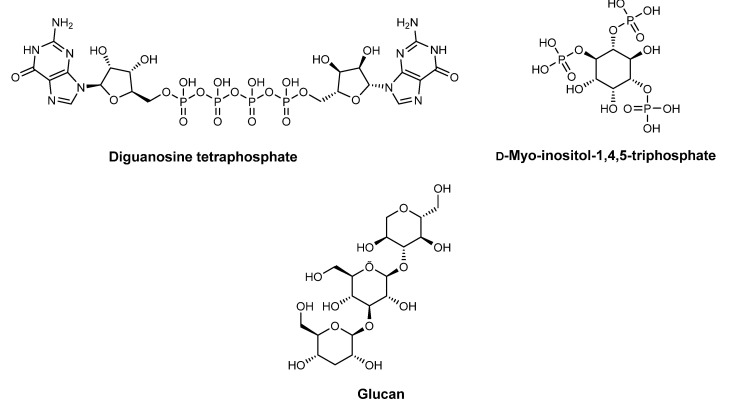
Maritime components of the tested cosmetic product containing *A. salina* extract.

Brine shrimp (*Artemia salina*, Linnaeus, 1758) is a simple marine invertebrate of about 1 mm in size. This animal is routinely used as live feed for marine larval fish since its freeze-dried cysts can last for several years and can be hatched into larvae without special equipment. Additionally, *A. salina* is widely used in the brine shrimp lethality assay (BSLA), a simple and inexpensive bioassay used for the preliminary assessment of toxicity. Extracts of *A. salina* have been largely explored for their anti-aging properties, which were previously reviewed by us [43]. Only a few in vivo studies were performed aiming to assess the efficacy of *Artemia* extracts as photoprotective agents. The use of a cosmetic product for the regeneration and stimulation of skin cells based on maritime components, comprised of (i) a product of an enzymatic extraction process of *A. salina* (phosphorylated nucleotides—diguanosine-tetraphosphate as the main component); (ii) D-myo-inositol-1,4,5-triphosphate; and (iii) glucan (Figure 14) (a:b:c ratio: 1:0.1–50:0.1–30) with additional cosmetic active ingredients and supporting materials, resulted in a synergetic effect against aggressive environmental influences by naturally strengthening the immune system of the skin, stimulating skin regeneration and, concomitantly, providing UV protection with the assistance of improved keratin barriers [25].

Hsp70, a heat shock protein, has protective effects against UV, apoptosis, and ischemia, through the inhibition of aggregation and assistance in the refolding of denatured proteins, and is recommended for wound healing and anti-aging purposes [141]. Studies on both cultured human epidermal cells and ex vivo skin showed that induction or administration of Hsp70 before stress significantly diminished UV-related morphological changes and sunburn cell number [26,27,28,142]. As an example, a study on the influence of heat shock proteins in the protection of cells from various types of stress has shown that treatment of human skin with *Artemia* extract induces Hsp70 in skin cells in a stress-free manner, enhancing aged skin defense from UV damage. The analysis of the punch biopsies from 10 volunteers showed a decrease in UV damage signs when compared to the untreated aged skin, confirming the fragility of aged skin towards UV stress and highlighting a new application of *Artemia* extract in the protection of aged skin [26]. Other similar studies involving the administration of *Artemia* extract were also reported with similar results [27,28,142].

*N. gaditana*

*N. gaditana* (L.M. Lubián, 1982) are mixotrophic, non-flagellated algae belonging to the class Eustigmatophyceae [143]. Several species belonging to the *Nannochloropsis* genus are sources of eicosapentaenoic acid (EPA), which is of nutritional importance [143]. Another highly interesting aspect is that *N. gaditana* produces lipids such as triacylglycerol (TAG), which is of special interest for human consumption since it has a composition of fatty acids similar to many vegetable oils [144]. Although the photoprotection strategies of the alga *N. gaditana* are well documented in the literature [145,146], only a limited number of studies have reported the beneficial role of this microalga in cosmetic formulations aimed to protect skin from UVR or aging factors. UVR (photoaging), oxidative stress (environmental pollution), and to a lesser extent, daily habits (smoking, poor nutrition) are the main extrinsic (external) factors responsible for the acceleration and extent of aging. Various strategies have been developed that can act synergistically with sunscreen filters and provide increased photoprotection in cosmetic formulations via various mechanisms. One of these strategies consists of the addition of active substances to cosmetic formulations that are commonly used for daily protection and care during low-sunshine periods. A combination of a *N. gaditana* extract and a propolis extract was tested as an active component in cosmetic formulations for dermal and in particular anti-aging use [147]. The protective role against the aging of human skin cells was confirmed by an increase in the ability of skin cells to produce collagen and elastin, by an increase in the expression of transcripts of fibrillin in skin cells, enhancing skin elasticity since this protein offers the scaffold for the proper conformation of elastin [147]. Another study to evaluate the effects of a *N. gaditana* extract against oxidative stress in human primary fibroblasts was performed to investigate the potential applications of this natural ingredient in cosmetics [143]. *N. gaditana* extract exhibited skin protection properties by mediating oxidative responses and apoptosis (including *SOD1*, *GPX1*, *BID*), positively regulating genes involved in skin texture and hydration (including *AQP3*, *Col6A1*, *FBN1*). Additionally, *N. gaditana* extract modulated the expression of genes involved in skin irritation, DNA damage and aging (including *IL1R*, *PCNA*, *FOXO3*).

*A. nodosum*

*A. nodosum* [(Linnaeus) Le Jolis, 1863] is a brown seaweed that belongs to the taxonomic order of Fucales. The characteristic brown color presented by these species results from the dominance of the pigment fucoxanthin, which masks the other pigments (chlorophyll a and c, β-carotene, and other carotenoids). Some of the most important constituents of this brown algae are phlorotannins, phloroglucinol-based polyphenols that are responsible for the well-known antioxidant activity exhibited by *A. nodosum* [20,148,149,150,151,152,153,154,155,156,157,158]. This brown alga has been highlighted in previous studies as a very promising ingredient due to its antioxidant properties, both in formulations for anti-aging [43] and sensitive skin [159]. Regarding photoprotection studies, some examples include a study of the seasonal and yearly variations in phenolic contents and associated cosmetic activities of seven brown marine macroalgae, the five studied Fucales (including *A. nodosum*) presented higher activities than Laminariales (IC_50_ = 0.010–0.353 mg·mL^−1^ (DPPH assay) and EC_50_ = 0.001–0.004 mg·mL^−1^ (FRAP assay)) with no significant differences between seasons or years for the different Fucales in either assay [20]. Concerning the FRAP assay, activities of fractions obtained from *A. nodosum* (spring 2016) were significantly higher than the vitamin C (*p* = 0.039) [20]. *A. nodosum* also revealed photoprotective activities since the photoprotective factors of the phlorotannin enriched fractions were greater than 1 (between 1.46 and 2.71 for SPF; 1.28 and 1.93 for PF-UVA), with the summer fraction showing the highest activity [20].

The photoprotective potential against UVB radiation of extracts obtained from 21 commercial macroalgae, including *A. nodosum*, was evaluated in vivo using the zebrafish embryo as a whole model organism [22]. *A. nodosum* showed an intermediate photoprotective capacity since 54% of the embryos showed a low level of malformations, but normal embryos were present in 8.3%. The authors hypothesized that phlorotannins are responsible for the “in vivo” photoprotection, supporting *A. nodosum*’s potential application in photoaging prevention [22].

*Spirulina platensis*

Spirulina, also known as *Arthrospira platensis* (Gomont, 1892), is an edible cyanobacteria (usually incorrectly classified as blue-green algae) that contains high levels of proteins, vitamins, fatty acids, and minerals and therefore is used as food, feed, and agricultural supplement [160]. Although numerous biological properties, such as antioxidant, anticancer, antimicrobial, and hepatoprotective effects, have been attributed to spirulina, the photoprotective effect of this alga was only recently explored [160,161]. Spirulina was also pointed to as a top ingredient to be included in anti-aging formulations, attributed mainly to its content of sulfated polysaccharides [43].

A recent study demonstrated for the first time that an extract of spirulina has a reverse effect on UV-induced photodamage such as loss of cell viability, cellular senescence, DNA damage, and collagen destruction in dermal fibroblasts [160]. The extract promoted G2/M cell cycle arrest and strongly inhibited UVB-induced cytotoxicity, senescence-associated β-galactosidase expression, and cyclobutane pyrimidine dimer formation in normal human dermal fibroblasts [160]. Additionally, the extract inhibited UVB-induced extracellular matrix degradation by downregulating matrix metalloproteinase 1 (MMP-1) and MMP-3 expression [160]. Another study explored the ability of spirulina to absorb the UVB spectra by determining its SPF, and by addressing its biological protective effects against UVB-irradiated human skin fibroblast cultures [161]. The photoprotective activity of spirulina was attributed to the presence of high amounts of phenolic compounds, producing a high degree of absorbance and yielding an SPF value of 30.39 [161]. No cytotoxicity was observed on UVB irradiation of human skin fibroblasts and the induction of the pro-inflammatory mediators IL-6 and IL-8 triggered by UVB irradiation was dramatically decreased after treatment with spirulina [161].

*A. armata*

*A. armata* (Harvey, 1855) is a red seaweed that can be found on European coasts and in the Northeast Atlantic [159]. Besides photosynthetic pigments, their main bioactive ingredients include agar, sulfated polysaccharides (carrageenans and porphyrans), and MAAs [159]. Two different studies aiming to screen selected red algae for the production of UV-screen substances, such as MAAs, identified shinorine at the highest concentration (about 80–85%), followed by palythine (17–20%) (Figure 13) and finally asterina-330 presenting only traces [23,162]. These low-molecular-weight water-soluble molecules present great potential as photoprotective and antioxidant ingredients since they can absorb UV radiation and disperse the absorbed energy as heat without generating reactive oxygen species (ROS) [159].

#### 2.2.3. Mechanisms of Action

There are several biological mechanisms of action by which these active ingredients could have beneficial effects; to disclose the molecular targets of the ingredients it is important to highlight the biological and cellular mechanisms associated with the UV-induced damage. The studied ingredients protect skin from a diverse range of adverse effects of solar UVR, including UVR-induced DNA damage/erythema, inflammation, photoaging, and the damage of free radicals produced by UVR exposure. It is noteworthy that all the biological mechanisms are strongly interconnected such as the UV-induced oxidative stress [163], associated with the excessive production of reactive species, namely ROS and RNS, which will contribute to triggering the expression of metalloproteinases [164]. Additionally, the activation of transcription factors such as NF-ĸB and the production of pro-inflammatory cytokines/interleukins are usually triggered by solar radiation, thus evoking inflammatory and immune responses [165,166]. Direct or indirect/oxidative damage in biomolecules, including cellular and mitochondrial DNA damage [167], crosslinking of extracellular matrix fibers, such as collagen, elastin, and fibronectin [168], oxidation of the skin lipids [169], and formation of reactive and toxic advanced glycation products (AGEs) in glycation process [170] are other harmful events of the solar-induced stress oxidative process. Dysregulation of melanogenesis, namely the activation of tyrosinase enzyme [171], and senescent events, including telomere shortening [172], may occur. The dual antioxidant-photoprotective activity of natural-derived compounds present in UV-photoprotective formulations, particularly botanical and marine ingredients, could be considered the gold pot for the development of novel and upgraded skincare products

### 2.3. Highlights

In this study, the prevalence of natural ingredients in the composition of sunscreen products was assessed. The following highlights illustrate a resume of the most important facts that could be drawn from this study:

- Two hundred and eleven (48%) of the 444 analyzed sunscreen formulations comprised ingredients from terrestrial plants, while marine ingredients were present in 57 (13%) of the studied formulations.
- The highest prevalence of ingredients from terrestrial plants might be attributed to their biodiversity, easy cultivation, and growth modulation, making them more attractive to the cosmetic industry.
- Twenty-nine (7%) of the total formulations contained both natural ingredients from terrestrial and marine environments, showing a possible synergism of metabolite action towards activity, without loss of the desired properties.
- The most used ingredients from terrestrial sources were obtained from the species *H. annuus*, *G. max*, *V. paradoxa*, *P. gratissima*, and *G. inflata*, all of which have been studied as photoprotective agents.
- The antioxidant and photoprotective activities of these extracts were mainly attributed to the presence of tocopherols, flavonoids, and phenolic acids.
- Algae, due to easy cultivation allied with the development of newer and improved techniques for their cultivation, constitute the most used marine ingredient, which is translated in the number of studies and scientific evidence available in the literature.
- Other marine ingredients such as *Artemia* or microorganisms were less used, probably due to the scarce scientific evidence of their photoprotective properties to back up their use in sunscreen formulations.
- The photoprotective effects of the analyzed marine ingredients are mainly attributed to MAAs. These low-molecular-weight water-soluble molecules proved to have anti-inflammatory effects by suppressing the expression of pro-inflammatory mediators in response to UV irradiation, therefore constituting a natural promising UV-absorbing alternative to common sunscreens.

## 3. Materials and Methods

### 3.1. Data Collection

The composition of a pool of skincare facial cosmetic products from multinational manufacturers, marketed in Portuguese parapharmacies and pharmacies was collected in 2021, to access the most used active marine and botanical ingredients in sunscreens. Sunscreen products with a labeled SPF were included in the study. All the information available in the product’s label was collected, along with the information available on the manufacturers’ websites.

### 3.2. Data Analysis

The natural ingredients contained in sunscreens were listed according to the International Nomenclature of Cosmetic Ingredients (INCI). Afterward, the data were analyzed concerning the following parameters:

#### 3.2.1. Natural Ingredients Use

The number of sunscreen products containing ingredients from terrestrial and/or marine sources was evaluated and expressed in percentage.

#### 3.2.2. Top Natural Ingredients Used in Sunscreens

The top ingredients from terrestrial and/or marine sources were identified from INCI lists and ranked in descending order of occurrence to disclose the top.

#### 3.2.3. Scientific Evidence Supporting the Efficacy of Natural Ingredients in Sunscreens

The efficacy data for each ingredient from terrestrial and/or marine sources were searched on the online databases PubMed, Scopus, Cochrane, KOSMET, and SciFinder^®^ [Chemical Abstracts Service (CAS)]. Due to the lack of studies regarding the applicability of active ingredients in sunscreens, a broader search was performed using the keywords “INCI name” OR “synonyms”, when applicable.

## 4. Conclusions

Over the last years, the search for valid alternatives of natural origin to complement the use of synthetic chemicals has been a growing trend in the cosmetic sector. As consumers become increasingly aware of the potential toxicity of synthetic cosmetic ingredients, products formulated with natural ingredients are increasingly reaching the consumer market. To date, an interesting number of ingredients from plants and marine organisms with photoprotective properties have already been incorporated in sunscreens that are currently on the market. Although some advances have been put towards the research and the use of ingredients from terrestrial plants in this type of formulation, the number of marine ingredients is still very minimal when compared to the botanical, especially considering the vastness of the sea and great potential for breakthroughs. Efforts should be deviated towards new strategies to increase the profitability of the extraction process, isolation and characterization of products from natural resources, and further investigation of their photoprotective potential and the safety profile regarding their application in the skin. However, some limitations are expected to avert the full exploitation of natural resources—the low concentrations of the active ingredient in the plant/organism responsible for the production of the ingredient, the variability on the environment (habitat, season of harvesting, and environmental conditions), which can cause different outcomes regarding their biosynthesis, and the fact that the mass cultivation of some species might endanger some already threatened ecosystems. The solution lies in finding sustainable ways to produce bioactive metabolites, paired with the optimization of growing and harvesting conditions to obtain enough high-valued compounds to be used as cosmetic ingredients.

Another important aspect lies with the fact that the efficacy and safety of some synthetic UV filters are hindered by their low photostability, and damage to marine ecosystems. The discovery that some of the chemicals found in sunscreens and other cosmetic products threaten the health of coral reefs entails a need for constant product development and reformulation. The photoprotective effects of natural ingredients are mainly exerted through their antioxidant effects, and through the regulation of UV-induced skin inflammation, barrier impairment, and aging. Even though natural ingredients cannot replace conventional UV filters, they can complement the photoprotective effectiveness afforded by organic and inorganic UV filters.

In conclusion, the use of natural ingredients in sunscreen formulations presents a great potential for the cosmetic industry, with a noticeable growing tendency over recent years. Nevertheless, the understanding of the photoprotection mechanisms used by natural organisms to bear sun overexposure should be further explored and extended to ensure the effectiveness and safety of the ingredients used for cosmetic application. This study provides an original and updated overview on the use of ingredients from plants and marine organisms in a panel of sunscreen formulations commercialized in the Portuguese market in 2021 and provides researchers and the cosmetic industry a perspective of the trends in the use of natural ingredients to develop new and improved formulations to prevent the deleterious effects of UVR.

## Figures and Tables

**Table 1:** Usage frequency of natural cosmetic ingredients from terrestrial sources—the top 10 species—and respective INCI and description.

Species	Usage (%)	INCI	Description
***H. annuus* (34)**			
*H. annuus* seed oil	34 (7.7%)	*H. annuus* seed oil	*H. Annuus* Seed Oil is the oil expressed from the seeds of the Sunflower, *H. annuus* L., Compositae
***Glycine max* (33)**			
*G. max* oil	30 (6.8%)	*Glycine soja* oil	*G. Soja* Oil is the oil obtained from the soybean, *G. soja*, Leguminosae, by extraction or expression. It consists essentially of triglycerides of oleic, linoleic and saturated acids
*G. max* seed extract	3 (0.7%)	*G. soja* seed extract	*G. Soja* Seed is an extract of the Soybean, *G. soja*, Leguminosae
***V. paradoxa* (26)**			
*V. paradoxa* butter	16 (3.6%)	*Butyrospermum parkii* butter	*B.Parkii* Seedcake Extract is the extract of the seedcake of the Shea Tree, *B. parkii*, Sapotaceae
*V. paradoxa* butter extract	8 (1.8%)	*B. parkii* butter extract	*B. Parkii* Butter is the fat obtained from the fruit of the Shea Tree, *B. parkii*, Sapotaceae
*V. paradoxa* butter seedcake extract	2 (0.5%)	*B. parkii* butter seedcake extract	*B. Parkii* Butter Extract is an extract obtained from the Shea Tree, *B. parkii*, Sapotaceae
***P. gratissima* (22)**			
*P. gratissima* fruit extract	11 (2.5%)	*P. gratissima* fruit extract	*P. Gratissima* Fruit Extract is an extract of the fruit of the Avocado, *P. gratissima*, Lauraceae
*P. gratissima* oil	11 (2.5%)	*P. gratissima* oil	*P. Gratissima* Oil is the fixed oil obtained by pressing the dehydrated sliced flesh of the avocado pear, *P. gratissima*, Lauraceae. It consists principally of the glycerides of fatty acids
***G. inflata* (21)**			
*G. inflata* root extract	21 (4.7%)	*G. inflata* root extract	*G. Inflata* Root Extract is an extract of the roots of *G. inflata*, Leguminosae
***T. parthenium* (16)**			
*T. parthenium* extract	7 (1.6%)	*Chrysanthemum parthenium* extract	*C. Parthenium* Extract is an extract of the herb of the feverfew, *C. parthenium*, Asteraceae
*T. parthenium* flower extract	9 (2.0%)	*C. parthenium* flower extract	*C. Parthenium* Flower Extract is an extract of the flowers the feverfew, *C. parthenium*, Asteraceae
***S. baicalensis* (16)**			
*S. baicalensis* extract	8 (1.8%)	*S. baicalensis* extract	*S. Baicalensis* Extract is the extract of the whole plant, *S. baicalensis*, Lamiaceae
*S. baicalensis* root extract	8 (1.8%)	*S. baicalensis* root extract	*S. Baicalensis* Root Extract is an extract of the roots of the *S. baicalensis*, Lamiaceae
***A. barbadensis* (15)**			
*A. barbadensis* leaf extract	2 (0.5%)	*A. barbadensis* leaf extract	*A. Barbadensis* Leaf Extract is an extract of the leaves of the aloe, *A. barbadensis*, Liliaceae
*A. barbadensis* leaf juice	5 (1.1%)	*A. barbadensis* leaf juice	*A. Barbadensis* Leaf Juice is the juice expressed from the leaves of the aloe, *A. barbadensis*, Liliaceae
*A. barbadensis* leaf juice powder	6 (1.4%)	*A. barbadensis* leaf juice powder	*A. Barbadensis* Leaf Juice Powder is the powder obtained from the dried juice leaves of the aloe, *A. barbadensis*, Liliaceae
*A. barbadensis* leaf water	2 (0.5%)	*A. barbadensis* leaf water	*A. Barbadensis* Leaf Water is an aqueous solution of the steam distillate obtained from the leaves of the aloe, *A. barbadensis*, Liliaceae
***C. nucifera* (14)**			
*C. nucifera* oil	14 (3.2%)	*C. nucifera* oil	*C. Nucifera* Oil is the fixed oil obtained by expression of the kernels of the seeds of the Coconut, *C. nucifera* L., Palmaceae
***P. leucotomos* (13)**			
*P. leucotomos* leaf extract	13 (2.9%)	*P. leucotomos* leaf extract	*P. Leucotomos* Leaf Extract is an extract of the leaves of *P. leucotomos*, Polypodiaceae

## Data Availability

Not applicable.
